# Respondent Driven Sampling: Determinants of Recruitment and a Method to Improve Point Estimation

**DOI:** 10.1371/journal.pone.0078402

**Published:** 2013-10-31

**Authors:** Nicky McCreesh, Andrew Copas, Janet Seeley, Lisa G. Johnston, Pam Sonnenberg, Richard J. Hayes, Simon D. W. Frost, Richard G. White

**Affiliations:** 1 Department of Infectious Disease Epidemiology, Faculty of Epidemiology & Population Health, London School of Hygiene and Tropical Medicine, London, United Kingdom; 2 School of Medicine, Pharmacy and Health, Durham University, Durham, United Kingdom; 3 Department of Infection and Population Health, University College London, London, United Kingdom; 4 MRC/UVRI Uganda Research Unit on AIDS, Entebbe, Uganda; 5 School of International Development, University of East Anglia, Norwich, United Kingdom; 6 Tulane University School of Public Health & Tropical Medicine, Department of International Health & Development, Center for Global Health Equity, New Orleans, Louisiana, United States of America; 7 Department of Veterinary Medicine, University of Cambridge, Cambridge, United Kingdom; Tulane University School of Public Health and Tropical Medicine, United States of America

## Abstract

**Introduction:**

Respondent-driven sampling (RDS) is a variant of a link-tracing design intended for generating unbiased estimates of the composition of hidden populations that typically involves giving participants several coupons to recruit their peers into the study. RDS may generate biased estimates if coupons are distributed non-randomly or if potential recruits present for interview non-randomly. We explore if biases detected in an RDS study were due to either of these mechanisms, and propose and apply weights to reduce bias due to non-random presentation for interview.

**Methods:**

Using data from the total population, and the population to whom recruiters offered their coupons, we explored how age and socioeconomic status were associated with being offered a coupon, and, if offered a coupon, with presenting for interview. Population proportions were estimated by weighting by the assumed inverse probabilities of being offered a coupon (as in existing RDS methods), and also of presentation for interview if offered a coupon by age and socioeconomic status group.

**Results:**

Younger men were under-recruited primarily because they were less likely to be offered coupons. The under-recruitment of higher socioeconomic status men was due in part to them being less likely to present for interview. Consistent with these findings, weighting for non-random presentation for interview by age and socioeconomic status group greatly improved the estimate of the proportion of men in the lowest socioeconomic group, reducing the root-mean-squared error of RDS estimates of socioeconomic status by 38%, but had little effect on estimates for age. The weighting also improved estimates for tribe and religion (reducing root-mean-squared-errors by 19–29%), but had little effect for sexual activity or HIV status.

**Conclusions:**

Data collected from recruiters on the characteristics of men to whom they offered coupons may be used to reduce bias in RDS studies. Further evaluation of this new method is required.

## Introduction

Hidden or hard-to-reach population subgroups, such as sex workers, often have an increased risk of certain infectious diseases such as HIV,[1]. It can be difficult to estimate the prevalence of infection and risk factors in these populations because of an inadequate sampling frame. A variety of convenience sampling techniques are typically used to collect data on these populations[2] however they cannot be used to generate unbiased population based estimates.

**Figure 1:**
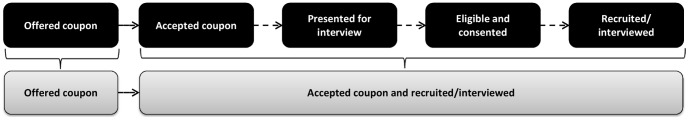
Diagram of the RDS recruitment process.

Respondent driven sampling (RDS)[3] is a variant of a link-tracing design that is designed to generate unbiased estimates of the prevalence of a disease or risk factors, or other characteristics, in a socially networked population. First, a small number of ‘seeds’ are selected by convenience. The seeds are given coupons, usually three, to recruit others from the target population. Recruits are given incentives both for taking part in the survey and for recruiting others. After their interview, the recruits are invited to become recruiters. If they accept, recruiters are asked to give coupons to other individuals in the target population with whom they have a relationship (Figure 1). To be recruited, individuals to whom coupons are offered need to accept, present for interview, be eligible and consent. They themselves can then become recruiters. This process continues in recruitment ‘waves’ until a target sample size is reached and/or until equilibrium is reached. Estimation methods are then applied to account for the non-random sample selection in an attempt to generate unbiased estimates for the composition of the target population. Two main estimation methods have been used, ‘RDS-1’, which accounts for patterns of recruitment between subgroups and the average number of other members of the target group recruiters know (the ‘network size’) in each subgroup[4], [5], and ‘RDS-2’, which, in its simplest formulation, accounts for network size only[6]. More recently, a more computationally intensive estimation method that relaxes some of the assumptions underlying the RDS-2 approach has been proposed[7].

Existing RDS estimation methods are designed to correct for varying probabilities of being offered a coupon, but only if these are distributed randomly, and are not designed to correct for bias arising if potential recruits accept coupons, and/or present for interview, non-randomly.

In a previous publication we reported on our study that evaluated whether RDS could generate representative data on 2402 male household heads in a rural Ugandan population-based cohort, by comparing estimates from an RDS survey with total-population data[8]–[10]. We found that both the sample proportions and the RDS-adjusted estimates were representative of the target population in most respects, but sample proportions and RDS-1 and RDS-2 point estimators under-estimated the proportions of younger men, men of higher socioeconomic status, men of unknown HIV status, and men with an unknown number of sexual partners in the population[8]. Neither RDS-1 nor RDS-2 adjustments improved the estimates, suggesting that the under-recruitment of these groups was not due to differences in network size.

Our analysis of qualitative data suggested potential explanations for the biases[8], [10]. Younger men may have been under-recruited because although they were defined as household heads by cohort field staff, they were not considered to be household heads by the community if they were unmarried and/or did not have children[8], [10]. Men of higher socioeconomic status may have been under-recruited because they were less attracted by the incentives for participation than men of lower socioeconomic status[8], [10].

In this study we explore the determinants of these biases in more detail, and attempt to elucidate if they were due mainly to men in these under-represented groups being less likely to be offered a coupon, or being less likely to present for interview if offered a coupon. We also propose and apply weighting for non-random presentation for interview as a method to reduce bias in RDS studies. Finally, we explore whether network size was predictive of recruitment.

## Methods

### Ethics statement

The Science and Ethics Committee of the Uganda Virus Research Institute (GC/l27109108), the Uganda National Council for Science and Technology (SS2278) and the London School of Hygiene and Tropical Medicine Ethics Committee (5585) gave ethical approval for the study. The Information sheet and consent form were translated into local languages. They were read out to the participants by the interviewer and discussed until the interviewer was confident that the information had been understood. Participants were asked to provide two copies of written informed consent. One copy was kept by the participant and the other was retained and archived by the MRC.

### Data

Full details of the survey population and survey methods are given in previous papers[8], [9]. We summarise key aspects below.

#### Target population

The target population for this study consisted of 2402 male household heads living in 25 villages in rural Uganda. The population of these villages make up an ongoing open cohort. The villages cover an area of approximately 38 km^2^ [11]. Each year, a total-population household census and an individual questionnaire and HIV-1 serosurvey were administered to the entire cohort. People were eligible for the respondent-driven sampling if they were recorded as a male head of a household during the household census between February 2009 and January 2010. Each household was required to identify one person as the head of household. Households were defined as a group of persons who normally live and eat together.

Data were available on the whole target population for tribe, religion, age, socio-economic status, number of sexual partners in the previous year (‘sexual activity level’), HIV status, and household socioeconomic status. Household socioeconomic status was calculated using principle components analysis from household ownership of 22 items recorded during an annual census (December 2008-October 2009) and categorised into quantiles based on the status of all households in the general population cohort villages. The exact locations of the houses of 2396 members of the target population were known. The direct distance (‘as the crow flies’) to the nearest interview site was calculated for these men.

#### RDS study

Ten seeds were purposively selected from the target population. 927 men (including the 10 seeds) were recruited. Seeds and recruits were offered incentives to the value of ∼$1US (1 kg soap, ½kg salt or four school notebooks) for participation and each recruitment. 75% of recruits (including seeds) (684) were offered coupons to recruit others, and of these 90% (612) accepted (called ‘recruiters’). 66% of recruiters (401) returned to collect their secondary incentives. This included 11%, 61%, 92%, and 99% of recruiters who had recruited zero, one, two, and three men respectively. These recruiters were interviewed for a second time and data were collected on whom they had offered coupons to. In total, they reported offering coupons to 1253 men. During the second interview with the recruiter, interviewers attempted to identify the men reported to have been offered coupons by comparing reported demographic information (name, village, tribe, religion and age) with a database of these details on all men seen in these communities by the Medical Research Council census staff. 847 men were identified, of whom 770 were members of the target population. 553 men were reported by one recruiter (only) as someone they had offered a coupon to, 93 by two recruiters, nine by three recruiters, and one by four recruiters. Overall, 27% (656/2402) of the target population were reported to have been offered a coupon by *at least* one recruiter, of who 77% (505/656) became recruits. The true proportion who were offered a coupon will be higher as not all recruiters returned to be interviewed for a second time. This also accounts for the fact only 55% (505/917) of the total recruits were reported to have been offered a coupon. In this paper, men were considered to have been ‘offered’ a coupon if they were reported as having accepted or refused a coupon by a recruiter. The method to determine recruits’ network sizes is shown in Methods S1.

To allow us to compare the characteristics of RDS recruits and non-recruits, immediately after the RDS survey, 300 men in the target population who had not been recruited into the RDS study were selected to be interviewed using simple random sampling. They were interviewed using the same questionnaire that was used with the RDS recruits during their first interview (see McCreesh *et al* [8] and Methods S1).

To help understand the quantitative study findings, 49 members of the population in the study villages or Medical Research Council staff were interviewed using qualitative methods(see McCreesh *et al* [10]).

### Hypotheses and data analysis strategy

#### Were RDS sample biases due to non-random coupon distribution or non-random presentation for interview?

Any under-recruitment of men with particular characteristics could have been because recruiters were less likely to offer coupons to these men, or because men with these characteristics were less likely to accept coupons and to present for interview, or through a combination of both mechanisms. Based on the findings from our earlier analysis[8], [10] summarized above, we hypothesized that the under-recruitment of younger men was mainly due to them having a lower probability of being offered a coupon (because they were not considered to be household heads by the community) and that the under-recruitment of higher socioeconomic status men was due mainly to them being less likely to accept coupons and/or being less likely to present for interview (because they were less attracted by the incentives).

We fitted two logistic regression models with the following outcome measures: (i) reported to have been offered a coupon (among the whole target population), and (ii) recruitment into the RDS study (only among those reported as having been offered a coupon). In both models we included age, socioeconomic status and participation in the last cohort round to see how these affect each outcome.

As only 66% of recruiters returned to answer questions on whom they offered coupons to, many men who were actually offered coupons will be classified by us as men who were not offered a coupon. If there is any difference in the proportion of men in each age or socioeconomic status group who were not reported to have been offered a coupon but who were in fact offered a coupon, then this may bias the findings of the analyses described in the previous paragraph. To examine whether this bias occurred, we compared the proportion of men who were reported to have been offered a coupon, by age and socioeconomic status group. We limited this analysis to men who were in the RDS sample, as we know that they must have been offered a coupon.

#### A method to improve point estimation: Interview presentation weighting

RDS-2 weighting obtains population estimates of population composition by weighting by the inverse of reported social network size (‘network size’ weights) We propose and apply a new weighting approach, which is based on RDS-2 weighting, but additionally tries to reduce bias due to differential acceptance of coupons and presenting for interview. We selected this bias because it should be possible to collect the data required for this adjustment relatively easily in a typical RDS study. The only additional data required are the visible characteristics of the individuals who were offered coupons. In our study, this data was collected from all members of the target population prior to the RDS study, however in a typical RDS study it would be collected from recruiters when they return for incentives. As an example of the logic, if we find that, *a*, the proportion of the final RDS sample in group *X* is smaller than, *b*, the proportion of all coupons offered to group *X*, then we know that if a coupon is offered to someone in group *X*, the probability of recruiting them is lower than if the same coupon had been offered to someone in the rest of the population(on average). As such, it is intuitive that our interview presentation weighting should increase the estimate of the size of group *X* relative to the rest of the population, to adjust for this bias.

As the characteristics of the coupon recipients need to be known by the recruiters in a typical RDS, we choose to apply this adjustment only to age and socioeconomic status, but not HIV and sexual activity (which is less likely to be known reliably by social contacts). Specifically, the adjustment was achieved by creating a second set of ‘interview presentation’ weights *w*, defined by 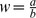, where *a* was the proportion of men in the RDS sample (excluding seeds) who were in the recruit's age and socioeconomic status groups and *b* was the proportion of all identified and eligible men in the population who were reported to have been offered a coupon who were in the recruit's age and socioeconomic status groups. For 3/917 recruits in 2/23 recruit age and socioeconomic status cross-classifications, there were no men who were reported to have been offered a coupon in the same age and socioeconomic status cross-classification. Recruits in these two cross-classifications were assigned recruitment probability weights of one. The inverse of the interview presentation weights, *w*, are then multiplied with the inverse network size weights (i.e. RDS-2 weights) to create a final set of interview presentation weights of the form 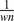 where *n* is reported network size. As such these weights can be seen as inverse-probability of interview presentation (i.e. coupon offered and accepted) weights, and as an example of inverse-probability weighting the approach has the same theoretical grounding as RDS-2 weighting. Population estimates for the characteristics age group, socioeconomic status, tribe, religion, sexual activity level, and HIV status, weighted by these interview presentation weights, were calculated. Root mean squared errors for the difference between the true population proportions and the estimated proportions were calculated each characteristic for the RDS-2 estimates and the interview presentation weighted RDS-2 estimates.

#### Relationship between network size and recruitment probability

We also explored the relationship between network size and recruitment probability. See Methods S1 for details.

## Results

### Were RDS sample biases due to non-random coupon distribution or non-random presentation for interview?

There was strong evidence that the odds of having been reported to have been offered a coupon reduced with decreasing age(Table 1). Compared to men aged 50+, men aged 40–49 had the same odds of having been reported to have been offered a coupon, men aged 30–39 had 0.71 times the odds, men aged 20–29 had 0.42 times the odds, and men aged <20 had 0.04 times the odds (p<0.0001, adjusting for socioeconomic status and participation in the last cohort round). However, if offered a coupon, there was no evidence for any difference in the odds of becoming a recruit by age (p = 0.4).

**Table 1:** Percentages, unadjusted and adjusted odds ratios (OR) for being reported to have been offered a coupon (N = 2402) and for being recruited if reported to have been offered a coupon (N = 656).

Variables	Category	Reported to have been offered a coupon (among whole target population) N = 2402			Recruited (among men reported to have been offered a coupon) N = 656		
		% (n/N)	Unadjusted OR (95% CI) (p-value)	Adjusted* OR (95% CI) (p-value)	% (n/N)	Unadjusted OR (95% CI) (p-value)	Adjusted* OR (95% CI) (p-value)
**Age**	**50+**	33% (236/714)	1	1	80% (189/236)	1	1
	**40–49**	32% (160/496)	0.96 (0.76–1.23)	1.00 (0.77–1.29)	77% (123/160)	0.83 (0.51–1.35)	0.88 (0.54–1.46)
	**30**–**39**	26% (170/660)	0.70 (0.56–0.89)	0.71 (0.56–0.91)	76% (129/170)	0.78 (0.49–1.26)	0.77 (0.47–1.25)
	**20**–**29**	18% (89/485)	0.46 (0.34–0.60)	0.42 (0.31–0.56)	71% (63/89)	0.60 (0.35–1.05)	0.61 (0.34–1.10)
	**<20**	2% (1/47)	0.04 (0.01–0.32)	0.04 (0.01–0.33)	100% (1/1)	-	
			(p<0.0001)	(p<0.0001)		(p = 0.4)	(p = 0.4)
**Socioeconomic status**	**Highest**	22% (134/617)	1	1	69% (93/134)	1	1
	**Higher**	28% (170/597)	1.44 (1.11–1.86)	1.40 (1.07–1.84)	76% (130/170)	1.43 (0.86–2.39)	1.64 (1.27–2.13)
	**Lower**	35% (190/550)	1.90 (1.47–2.47)	1.83 (1.39–2.39)	78% (149/190)	1.60 (0.97–2.65)	2.32 (1.78–3.02)
	**Lowest**	27% (139/514)	1.34 (1.02–1.76)	1.17 (0.88–1.56)	85% (118/139)	2.48 (1.37–4.48)	2.27 (1.73–2.96)
	**Unknown**	19% (23/124)	0.82 (0.5–1.34)	0.97 (0.58–1.61)	65% (15/23)	0.83 (0.33–2.10)	1.34 (0.85–2.12)
			(p<0.0001)	(p = 0.0001)		(p = 0.02)	(p = 0.06)
**Participated in last cohort round**	**No**	17% (190/1114)	1	1	65% (123/190)	1	1
	**Yes**	36% (466/1288)	2.76 (2.27–3.34)	2.84 (2.33–3.46)	82% (382/466)	2.48 (1.69–3.62)	4.30 (3.57–5.19)
			(p<0.0001)	(p<0.0001)		(p<0.0001)	(p<0.0001)

P-values are overall p-values for the association. *  =  Adjusted for other variables in the table. The order of the age categories has been reversed so that 50+ year olds are the reference category due to the small number of <20 year old men who were reported to have been offered a coupon

The odds of having been reported to have been offered a coupon were lowest in men in the highest socioeconomic status group and in men of unknown socioeconomic status(Table 1). Compared to men in the highest socioeconomic status group, men in the higher group had 1.40 times the odds of having been reported to have been offered a coupon, men in the lower group had 1.83 times the odds, men in the lowest group had 1.17 times the odds, and men of unknown socioeconomic status had 0.97 times the odds (p<0.0001, adjusting for age and participation in the last cohort round). The odds of recruitment among men who were reported to have been offered a coupon increased with decreasing socioeconomic status, and were lowest in men of unknown status. Compared to men in the highest socioeconomic status group, men in the higher group had 1.64 times the odds of recruitment, men in the lower group had 2.32 times the odds, men in the lowest group had 2.27 times the odds, and men of unknown status had 1.34 times the odds (p = 0.06, adjusting for age and participation in the last cohort round).

It is possible that the differences in the odds of recruitment by socioeconomic status among men who were reported to have been offered a coupon were not due to differences in the odds of someone who had been offered a coupon accepting it and presenting for interview, but instead due to bias in the reporting of coupon offering. This was assessed by comparing the proportions of RDS recruits in each age group and socioeconomic status group who were reported to have been offered a coupon. There was no evidence for any difference in the proportions by age group (p = 0.7) but some evidence for a difference by socioeconomic status(p = 0.05)(Table 2).

**Table 2:** Associations with being reported to have been offered a coupon among RDS recruits (N = 917).

		Number reported to have been offered a coupon	% (p-value)
**Age**	**0**–**19**	1/4	25%
	**20**–**29**	63/122	52%
	**30**–**39**	129/229	56% (p = 0.7)
	**40**–**49**	123/220	56%
	**50+**	189/342	55%
**Socioeconomic status**	**Highest**	93/164	57%
	**Higher**	130/222	59%
	**Lower**	149/252	59% (p = 0.05)
	**Lowest**	118/244	48%
	**Unknown**	15/35	43%
**Participated in last cohort round**	**No**	123/238	52% (p = 0.2)
	**Yes**	382/679	56%

P-values are overall p-values for the association. Recruits were reported to have been offered a coupon if one or more recruiters said that they had offered them a coupon.

### A method to improve point estimation: Interview presentation weighting

Interview presentation weighting, which was designed to correct for bias in the acceptance of coupons, reduced the root mean squared error of the RDS-2 estimates for socioeconomic status by 38% (0.056 to 0.034), for tribe by 29% (0.026 to 0.019) and for religion by 19% (0.029 to 0.023), but had little effect on the RDS-2 estimates for age group (a decrease in root mean squared error of 2% (0.058 to 0.057)), sexual activity level (increase of 1.8% (0.122 to 0.124)), and HIV status (increase of 0.4% (0.1842 to 0.1843))(Table 3).

**Table 3:** Comparison of standard RDS-2 estimates and interview presentation weighted RDS-2 estimates (the latter adjusts for non-random presentation for interview if offered a coupon by age and socioeconomic status).

		Proportions			Estimates		Root mean squared errors	
		Population	RDS sample	Men reported to have been offered a coupon	RDS-2	Interview presentation weighted RDS-2	RDS-2 estimates	Interview presentation weighted RDS-2 estimates
**Age group (years)**	**0**–**19**	2.0%	0.4%	0.1%	0.5%	0.6%		
	**20**–**29**	20.2%	13.3%	12.7%	12.9%	12.1%		
	**30**–**39**	27.5%	25.0%	24.9%	24.2%	24.6%	0.058	0.057
	**40**–**49**	20.7%	24.0%	25.6%	23.0%	24.7%		
	**50+**	29.7%	37.3%	36.6%	39.5%	37.9%		
**Socioeconomic status**	**Highest**	25.7%	17.9%	21.3%	17.0%	20.5%		
	**Higher**	24.9%	24.2%	26.1%	23.8%	26.1%		
	**Lower**	22.9%	27.5%	28.6%	26.9%	28.0%	0.056	0.034
	**Lowest**	21.4%	26.6%	20.6%	29.0%	22.3%		
	**Unknown**	5.2%	3.8%	3.4%	3.3%	3.2%		
**Tribe**	**Muganda**	69.7%	66.7%	70.4%	66.1%	67.5%		
	**M'rwanda/kole**	17.9%	21.0%	18.5%	21.2%	20.4%		
	**Mukiga**	1.7%	2.1%	1.4%	2.2%	2.2%	0.026	0.019
	**Murundi**	4.7%	6.1%	6.6%	6.9%	6.1%		
	**Other known/unknown**	6.0%	4.0%	3.2%	3.6%	3.8%		
**Religion**	**Catholic**	59.8%	62.4%	61.1%	64.5%	63.6%		
	**Protestant**	17.0%	17.1%	17.8%	15.3%	15.1%	0.029	0.023
	**Muslim**	22.7%	20.2%	20.9%	19.8%	20.8%		
	**Other known/none/unknown**	0.5%	0.3%	0.2%	0.4%	0.6%		
**Number of sexual partners in the last year**	**0**	11.3%	14.8%	12.0%	16.1%	13.7%		
	**1**	41.9%	57.7%	57.0%	57.4%	58.9%		
	**2**–**3**	11.4%	14.0%	13.9%	12.8%	13.4%	0.122	0.124
	**4+**	3.7%	3.5%	5.3%	4.0%	4.0%		
	**Unknown**	31.6%	10.0%	11.7%	9.8%	10.0%		
**HIV status**	**Positive**	6.3%	7.9%	7.2%	7.4%	7.0%		
	**Negative**	60.0%	81.7%	78.1%	82.0%	82.2%	0.184	0.184
	**Unknown**	33.7%	10.5%	14.8%	10.6%	10.8%		

Root mean squared errors calculated relative to population proportions

### Relationship between network size and recruitment probability

**Figure 2:**
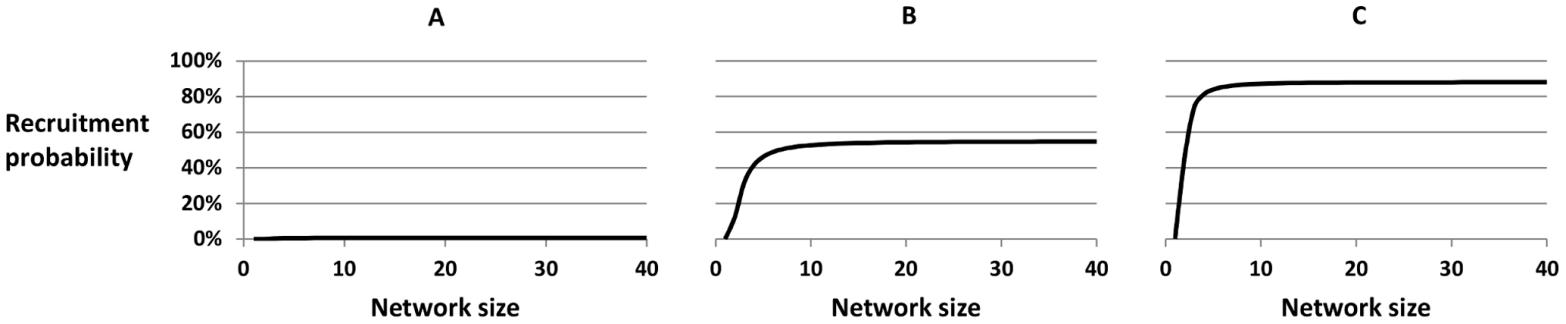
Relationship between network size and recruitment probability in the target population. The relationship is shown for three men with (A) low, (B) medium, and (C) high probabilities of recruitment based on the other variables in the logistic regression model (age group, socioeconomic status, tribe, hiv status, sexual activity group, distance to the nearest interview site and participation in the last cohort round. The figure shows that the relationship is non-linear, and that network size has little effect on recruitment probability apart from for men with small network sizes.

The overall mean network size was 7.4 for the simple random sample of RDS non-recruits and 12.1 for RDS recruits, and the estimated mean network size for the target population was 9.2 (the weighted mean of the mean for the simple random sample of RDS non-recruits and the mean for the RDS recruits). The adjusted mean network size for the target population was 9.9 (calculated from the network size data from RDS recruits only, assuming a linear relationship between inverse network size and recruitment probability). The fractional polynomial analysis showed that the best relationship between recruitment probability and network size (*n*) was not 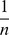 but 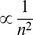. 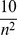 was used as our transformed network size, as opposed to 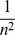, to make the odds ratio for transformed network size easier to interpret. The odds of recruitment decreased by 54% for every increase of one in 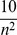 (Table 4). Figure 2 illustrates this by showing how the recruitment probability varied with network size for three men with low, medium and high probability of recruitment (based on the other variables in the model) for this best fitting relationship.

**Table 4:** Logistic regression model for recruitment into the RDS study.

Variable	Category	Adjusted* OR	95% Confidence interval
**Age (years)**	**<20**	1	
	**20**–**29**	2.81	1.03–7.65
	**30**–**39**	5.37	1.97–14.49
	**40**–**49**	8.84	3.23–24.02
	**50+**	9.33	3.43–25.13
**Socioeconomic status**	**Highest**	1	
	**Higher**	1.77	1.34–2.34
	**Lower**	2.32	1.74–3.07
	**Lowest**	2.55	1.89–3.44
	**Unknown**	1.52	0.92–2.52
**Tribe**	**Ganda**	1	
	**Rwanda/kole**	1.12	0.87–1.44
	**Kiga**	1.65	0.80–3.41
	**Rundi**	1.43	0.91–2.24
	**Other known/unknown**	0.67	0.43–1.06
**HIV status**	**Negative**	1	
	**Positive**	1.14	0.78–1.66
	**Unknown**	0.28	0.21–0.37
**Number of sex partners in the past year**	**0**	1	
	**1**	1.75	1.27–2.40
	**2**–**3**	1.48	1.00–2.19
	**4+**	1.01	0.58–1.75
	**Unknown**	0.40	0.26–0.60
**Distance to the nearest interview site (km)**	**0**–**1**	1	
	**1**–**2**	0.50	0.37–0.66
	**2**–**3**	0.50	0.37–0.67
	**3+**	0.42	0.28–0.64
**Participated in the last cohort round**	**No**	1	
	**Yes**	1.22	0.93–1.59
Transformed network size **		0.46	0.22–1.01

N = 2396 due to missing data for the variable distance to the nearest interview site. *  =  Adjusted for other variables in model. ** Transformed network size  = 
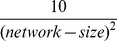
.

## Discussion

There was strong evidence that younger men were less likely to have been reported to have been offered a coupon. In contrast, there was no evidence that younger men were less likely to be recruited if they were reported as being offered a coupon. This suggests that the under-recruitment of younger men was largely because they were less likely to have been offered a coupon. There was no evidence for any difference in the proportion of recruits in each age group who were reported to have been offered a coupon, and therefore our findings are unlikely to be due to bias in whom recruiters reported offering coupons.

There was strong evidence that men in the highest socioeconomic status group and men of unknown socioeconomic status were less likely to have been reported to have been offered a coupon. However, in contrast to age, there was also evidence that men of higher socioeconomic status were less likely to have been recruited if they were offered a coupon. This suggests that at least some of the under-recruitment of higher socioeconomic status men was because they were less likely to present for interview if offered a coupon.

As such, we expected our newly proposed interview presentation weights to reduce the bias on socioeconomic status more than the bias on age (because the weights were designed to reduce bias due to differential presentation for interview among those offered a coupon). Indeed we found this to be correct with the root mean squared error reduced more for socioeconomic status (38%), than age (2%). The improvement for socioeconomic status was largely due to a greatly improved estimate of the proportion of men in the lowest socioeconomic status group. The interview presentation weights also improved estimates for tribe and religion (reducing root mean squared errors by 29% and 19% respectively), but had little effect on estimated for sexual activity level or HIV status (increasing root mean squared errors by 1.8% and 0.04% respectively).

As we showed earlier[8], weighting by the inverse of network size to produce RDS-1 or RDS-2 estimates did not improve estimates of population proportions. This analysis suggests that the observed non-linear relationship between inverse network size and recruitment probability (Figure 2) may explain why, as RDS theory and adjustments assume that the relationship is linear. As our findings show, this assumption was not true in this population, and therefore weighting by the inverse of network size (as in RDS-2 weighting) was not appropriate. This may apply to other RDS studies, and has also been demonstrated through theoretical work, leading to a further recent estimator[7], [12].

We illustrated our interview presentation weighting procedure by combining the inverse interview presentation (once offered a coupon) probability weights with the inverse coupon offer weights from the RDS-2 approach, which is simple to understand. The ability of our new method to reduce bias will therefore have been constrained by the failure of the assumptions underlying RDS-2, and our results may not apply to other estimators. Our method could potentially be used with inverse coupon offer probability weights from any approach however, and is therefore not restricted to RDS-2, and an interesting though challenging possibility is to try to develop these weights from modeling how participant characteristics are associated with subsequent recruitment effectiveness and speed. Further studies could examine the performance of estimates generated by combining inverse interview presentation probability weights with the weights from this or from recent approaches[7].

Another area for further work is in variance estimation, which is challenging in RDS studies. We have suggested applying weights for non-random interview presentation through splitting participants into distinct strata within which these weights are constant. Hence if the variance of an estimator (allowing for non-random coupon offer) can be estimated within each stratum then the variance of the estimator we propose can also be estimated following the ideas of post-stratification in survey analysis[13]. The additional weighting we propose will likely increase the variance of estimators, though this is offset by an expected reduction in bias. As improved point and variance estimators are developed for RDS samples it will be beneficial to present how these can be adapted to deal with non-random interview presentation following the approach we have outlined here.

Our results need to be interpreted with caution for at least three reasons. First, although it is plausible that higher socioeconomic status men were less motivated by the incentive or were under less social pressure to go for recruitment if offered a coupon, our finding may also have been due to bias in the reporting of who was given coupons(Table 2). Second, only 66% of recruiters returned for their follow-up interview[8]. We are therefore likely to be missing data on some men who were offered a coupon but did not present for interview. The effects of differential non-response by the variable of interest could be larger than the effects of non-response in a standard sampling design. As men who did not return for the second interview recruited fewer people on average than men who did return it is possible that they offered coupons to a higher proportion of people in groups that were less likely to present for interview if offered a coupon. This will have reduced the ability of our weighting method to fully correct for interview-presentation bias. Offering an additional incentive to recruiters for returning for a second interview after a suitable period of time, regardless of whether or not they have recruited anyone, could greatly reduce loss to follow-up and improve the performance of the estimator. Third, unknown network sizes were imputed for RDS-non-recruits. As network size data were collected from a random sample of eligible non-RDS-recruits, there should be no systematic difference between the missing and known network sizes of RDS-non-recruits. There was a low response rate among non-RDS-recruits selected for interview however, and therefore imputation may have given misleading results and the true relationship between network size and recruitment probability may have been distorted. Nevertheless, given the strongly non-linear relationship found by this study, it is unlikely that the true relationship was linear.

There are two ways in which recruitment bias can occur in RDS studies. The first is if recruiters are less likely to offer coupons to certain groups of people. In our study this bias resulted in the under-recruitment of younger men and is likely to have occurred because the study population and survey researchers held different definitions of the target population (household heads). RDS surveys are more susceptible to this type of bias than most surveys, because in RDS studies members of the study population carry out the selection of participants. To prevent this from happening in future RDS studies, we suggest that in addition to explaining the study inclusion and exclusion criteria to all potential recruiters, field-staff also actively test the recruiters' understanding of these criteria before coupons are released. Formative research is also important, and if understanding proves unreliable during piloting, it may be preferable to use a wider definition for recruitment that more closely matches a definition already used by the community, and only apply the narrower target group definition during analysis. Qualitative research conducted throughout the implementation of the study may also identify problems as they occur.

The second way that recruitment bias can occur in RDS studies is if certain groups are less likely to accept coupons or present for interview after having been offered a coupon than others. This occurred with higher socioeconomic status men in our study, most likely because they were less attracted by the incentives offered. This is a problem which is liable to arise in most, if not all, RDS studies. It may be possible to reduce this bias in some cases by increasing the size of the incentive offered, but this may also result in an increase in the number of non-eligible people attempting to take part in the study by deceiving the researchers. We have demonstrated that it is possible to reduce this form of bias and improve RDS estimates using data collected from recruiters returning to obtain their secondary incentives. We have also demonstrated that it is possible to reduce bias in variables other than the ones on which data are collected. We used accurate data on members of the target population collected before the RDS study. In a typical RDS study however, this information would need to be collected directly from recruiters. As such it is likely to be most successful at improving estimates of characteristics that tend to be visible (eg age or gender) or characteristics that are associated with visible characteristics (eg religion and tribe in our study). If the information is sufficiently accurate, then it will be possible to use the method shown in this paper to reduce recruitment bias. If the information obtained is less detailed or accurate, then it may still be used to assess the likelihood that this form of recruitment bias occurred. It should be noted that the ability of the method to improve estimates will be reduced if recruiters decide not to offer coupons to individuals who they think are unlikely to present for interview.

Further studies examining the relationship between network size and recruitment probability into RDS studies, and the new recruitment probability weights, would be useful.

## Supporting Information

Methods S1 Information on network size calculation, the simple random survey, and the relationship between network size and recruitment probability.
(DOCX)
