# Supplementary material for: Respondent Driven Sampling: Determinants of Recruitment and a Method to Improve Point Estimation
Source: PLoS One. 2013 Oct 31;8(10):e78402. doi: 10.1371/journal.pone.0078402 (PMC3814964; doi:10.1371/journal.pone.0078402)
Supplement: Methods S1 — Information on network size calculation, the simple random survey, and the relationship between network size and recruitment probability. (DOCX) [file pone.0078402.s001.docx]

**Supporting methods S1**

**Methods: Method to determine recruits’ network sizes**

Recruits’ network sizes were determined from their response to the question: “*How many men do you know who (i) were head of a household in the last 12 months in any of the Medical Research Council villages, and (ii) you know them and they know you, and (iii) you have seen them in the past week*?”, which was asked during their first interview.

**Methods: Simple random sampling survey.**

To allow us to compare the characteristics of RDS recruits and non-recruits, immediately after the RDS survey, 300 men in the target population who had not been recruited into the RDS study were selected to be interviewed using simple random sampling. They were interviewed using the same questionnaire that was used with the RDS recruits during their first interview. 54% (162/300) of the men selected were interviewed. The response rate was higher among men who had participated in the last cohort round than among men who had not participated (61% [78/127] vs 50% [86/173], p=0.04) (see McCreesh *et al*[*^1^*](#_ENREF_1)).

**Methods: Relationship between network size and recruitment probability**

To explore the relationship between network size and recruitment probability, the mean network size was calculated for the simple random sample of non-recruits and for the RDS sample. The mean network size for the whole target population was calculated as the weighted average of the mean network sizes of the simple random sample and RDS recruits. The ‘adjusted mean network size’ for the RDS sample was also calculated. This is a measure which assumes the mean network size for the target population from the RDS sample data only, assuming that men with higher network sizes are more likely to be recruited[^2^](#_ENREF_2).

Network size was unknown for 55% of the target population(1314/2402) who were not interviewed as part of the RDS study or as part of the simple random sample. Network size was imputed for these men, using multiple imputation using the Stata 11[^3^](#_ENREF_3) command ‘*mi impute*’ based on a linear regression model including as covariates the variables age group, socioeconomic status, tribe, HIV status, sexual activity level, participation in the last cohort round, and participation in the RDS study. 50 imputed datasets were created, and combined using Rubin’s formula[^4^](#_ENREF_4).

Standard RDS theory assumes a linear relationship between inverse network size and recruitment probability. We explored the possibility that the relationship between network size and log odds of the recruitment into our RDS survey was not linear by using fractional polynomial analysis [^5^](#_ENREF_5)^,^[^6^](#_ENREF_6). We explored non-linearity by fitting first and second-order fractional polynomial transformations of the network size ($n$), to the log odds of recruitment. The best power transformation $n^{p}$ was defined as that which resulted in the smallest model deviance with a $X^{2}$ distribution on 1 degree of freedom, assuming a 5% level for significance testing. The candidate set includes the linear relationship ($p=1$), and reciprocal, logarithmic, square root, and square transformations. $p$ was chosen from candidates -2, -1, -0.5, 0, 0.5, 1, 2, 3, where $n^{0}$ represents $log n$. For example for $p=-1$, the model is $b_{0}+ \frac{b_{1}}{n}$. Imputed values of network size were included. This transformed network size was then included in a logistic regression model for recruitment into the RDS study. Graphs showing how the recruitment probability varied with network size for three men with low, medium and high probability of recruitment(based on the other variables in the model) were produced to illustrate the best fit relationship.

**Supporting References**

References

1. McCreesh N, Frost SDW, Seeley J, Katongole J, Tarsh MN, Ndunguse R, Jichi F, Lunel NL, Maher D, Johnston LG, Sonnenberg P, Copas AJ, Hayes RJ, White RG. Evaluation of Respondent-driven Sampling. *Epidemiology* 2012;**23**(1):138-147 10.1097/EDE.0b013e31823ac17c.

2. Heckathorn DD. Extensions of Respondent-Driven Sampling: Analyzing Continuous Variables and Controlling for Differential Recruitment. *Sociological Methodology* 2007;**37**(1):151-207.

3. StataCorp. Stata Statistical Software: Release 11.0. 9 ed. College Station, Texas: Stata Press, 2010.

4. Rubin D. *Multiple Imputation for Nonresponse in Surveys* John Wiley & Sons., 1987.

5. Royston P, Ambler G, Sauerbrei W. The use of fractional polynomials to model continuous risk variables in epidemiology. *Int J Epidemiol* 1999;**28**(5):964-74.

6. Royston P, Altman DG. Regression using fractional polynomials of continuous covariates: parsimonious parametric modelling. *Applied Statistics* 1994:429-467.
